# Effects of APOE ε2 on the Fractional Amplitude of Low-Frequency Fluctuation in Mild Cognitive Impairment: A Study Based on the Resting-State Functional MRI

**DOI:** 10.3389/fnagi.2021.591347

**Published:** 2021-04-29

**Authors:** Xiaocao Liu, Qingze Zeng, Xiao Luo, Kaicheng Li, Hui Hong, Shuyue Wang, Xiaojun Guan, Jingjing Wu, Ruiting Zhang, Tianyi Zhang, Zheyu Li, Yanv Fu, Tao Wang, Chao Wang, Xiaojun Xu, Peiyu Huang, Minming Zhang, for the Alzheimer’s Disease Neuroimaging Initiative (ADNI)

**Affiliations:** ^1^Department of Radiology, The 2nd Affiliated Hospital of Zhejiang University School of Medicine, Hangzhou, China; ^2^Department of Neurology, The 2nd Affiliated Hospital of Zhejiang University School of Medicine, Hangzhou, China

**Keywords:** resting-state functional MRI, APOE, Alzheimer’s disease, mild cognitive impairment, fractional amplitude of low-frequency fluctuation

## Abstract

**Background:**

Apolipoprotein E (APOE) ε2 is a protective genetic factor for Alzheimer’s disease (AD). However, the potential interaction effects between the APOE ε2 allele and disease status on the intrinsic brain activity remain elusive.

**Methods:**

We identified 73 healthy control (HC) with APOE ε3/ε3, 61 mild cognitive impairment (MCI) subjects with APOE ε3/ε3, 24 HC with APOE ε2/ε3, and 10 MCI subjects with APOE ε2/ε3 from the ADNI database. All subjects underwent a resting-state functional MRI and Fluoro-deoxy-glucose positron emission tomography (FDG-PET). We used a fractional amplitude of low-frequency fluctuation (fALFF) to explore the spontaneous brain activity. Based on the mixed-effects analysis, we explored the interaction effects between the APOE ε2 allele versus disease status on brain activity and metabolism in a voxel-wise fashion (GRF corrected, *p* < 0.01), followed by *post hoc* two-sample *t*-tests (Bonferroni corrected, *p* < 0.05). We then investigated the relationship between the mean imaging metrics and cognitive abilities.

**Results:**

There are no significant differences in gender, age, or education among the four groups. The interaction effect on brain activity was located in the inferior parietal lobule (IPL). *Post hoc* analysis showed that APOE ε2/ε3 MCI had an increased IPL fALFF than APOE ε3/ε3 MCI. Regarding the APOE ε2 allele effects, we found that ε2 carriers had a decreased fALFF in the transverse temporal gyrus than non-carriers. Also, FDG-PET results showed a lower SUVR of the frontal lobe in APOE ε2 carriers than non-carriers. Furthermore, fALFF of IPL was correlated with the visuospatial function (*r* = −0.16, *p* < 0.05).

**Conclusion:**

APOE ε2 carriers might have a better brain reservation when coping with AD-related pathologies.

## Introduction

Alzheimer’s disease (AD) is the most common cause of dementia and is usually clinically characterized by a progressive and irreversible cognitive decline. Neuropathological alterations in AD involve the extracellular β-amyloid deposits and intraneuronal neurofibrillary tangles (Braak and Braak, 1991). Considering that AD-related pathology gradually accumulates in the brain even more than 10 years before the disease onset, more studies focused on the prodromal stages of AD could deepen our understanding of the disease continuum. Accordingly, as a transitional stage between healthy aging and AD, mild cognitive impairment (MCI) has been an area of significant interest in the last decades (Grundman et al., 2004; Petersen and Negash, 2008; Albert et al., 2011). Owing to the heterogeneous nature of MCI, exploring the neuropathological mechanisms behind a high or low-risk MCI may facilitate an earlier diagnosis and timely intervention (Vuoksimaa et al., 2018).

Multiple factors contribute to the onset of AD (Jack et al., 2018). Notably, apolipoprotein E (APOE, gene) is one of the most influential genetic factors for sporadic AD (Liu et al., 2013). Specifically, APOE ε4 and ε2 are risks and protective factors for AD, respectively (Corder et al., 1993; McKenna et al., 2016). However, APOE ε2 is previously overlooked, but critical to risk stratification (Suri et al., 2013). Past epidemiological studies revealed that the risk for APOE ε2 homozygous carriers to develop AD is four times lower than that of a healthy elderly carrying APOE ε4 (Corder et al., 1994). Furthermore, APOE ε2 carriers had a lower decline rate in episodic memory (Helkala et al., 1996), fewer Aβ plaques accumulation, and more effective Aβ clearance (Lippa et al., 1997; Tiraboschi et al., 2004; Sharman et al., 2010) than ε3 homozygous. While accumulated evidence shows that the APOE ε2 allele plays a protective role in AD, some studies proposed relatively conservative ideas. Berlau et al. indicated that APOE ε2 is associated with an intact cognition but increased Alzheimer’s pathology in the oldest old (Berlau et al., 2009). One pathological study showed that the APOE ε2 allele was associated with large but circumscribed protective effects (Goldberg et al., 2020). Also, the APOE ε2 appears to have a relatively selective impact on reduced pathology in the aged brain (Grothe et al., 2017). Chen et al. suggested that the ε2 allele plays a pivotal role in compensating for worsening neuropathological changes in an amnestic MCI (aMCI; Chen et al., 2016).

Detection of regional abnormalities is crucial to clinical studies and even clinical applications. However, the APOE ε2 allele’s effects on regional abnormalities in disease development progression are still unclear. As we know, resting-state functional magnetic resonance imaging (rs-fMRI) evaluates the spontaneous fluctuations of blood oxygenation level-dependent (BOLD) signals in different brain regions without performing specific behavioral or cognitive tasks. One of the imaging metrics for rs-fMRI is the fractional amplitude of low-frequency fluctuations (fALFF), which detected a regional spontaneous brain activity with sensitivity and specificity. They also indicated that the default mode network is reputably detected using fALFF (Zou et al., 2008). Some groups have also demonstrated an abnormal fALFF in brain diseases such as AD (He et al., 2007), schizophrenia (Hoptman et al., 2010; Sui et al., 2015), and epilepsy (Pedersen et al., 2016; Reyes et al., 2016). However, it is mostly unknown whether APOE ε2 carriers show abnormal changes in the regional brain activity. On the other hand, Fluoro-deoxy-glucose positron emission tomography (FDG-PET) could measure the brain’s metabolic status by examining the values of cerebral metabolism (Grady et al., 2003). Drzezga et al. demonstrated that 18F-FDG-PET was a reliable diagnostic tool for predicting individual MCI patients (Drzezga et al., 2005). Precisely, FDG-PET measures the mean glucose metabolism over minutes, while rs-fMRI evaluates the dynamic characteristics (Jiao et al., 2019) and the low-frequency (0.01–0.08 Hz) fluctuations (LFFs) in the fMRI of blood-oxygen-level-dependent (BOLD) fMRI signals which are related to the spontaneous neuronal activities (Logothetis et al., 2001; Goldman et al., 2002; Lu et al., 2007; Mantini et al., 2007). The combination of the two methods is complementary to explore the change in the local brain activity.

The study aimed to utilize fALFF to examine the APOE ε2 allele related changes in the earlier stages with AD in regional spontaneous brain activity. Based on previous reviews (Zou et al., 2008; Berlau et al., 2009; Chen et al., 2016; Grothe et al., 2017; Goldberg et al., 2020), the APOE ε2 allele is closely related to the disease progression and may be associated with limited protection. We hypothesize that (1) there is an interactive effect between APOE ε2 and disease conditions and (2) ε2 carriers require a lower brain activity than non-carriers to maintain normal brain functions after pathological deposition.

## Materials and Methods

### Study Participants

The data used in the study was obtained from the Alzheimer’s disease Neuroimaging Initiative (ADNI) database.^1^ This study was approved by the Institutional Review Boards of all of the participating institutions, and informed written consent was obtained from all participants at each site. At the time of analysis, we divided the subjects into four groups, namely, MCI with APOE ε3/ε3, MCI with APOE ε2/ε3, healthy controls (HC) with APOE ε3/ε3, and HC with APOE ε2/ε3. A total of 446 right-handed participants who had undergone structural scans, rsfMRI scans, and neuropsychological evaluations, comprised of 133 MCI with APOE ε3/ε3, 16 MCI with APOE ε2/ε3, 253 HC with APOE ε3/ε3, and 94 HC with APOE ε2/ε3, were identified from the ADNI GO, ADNI 2, and ADNI 3 databases. Imaging data and demographics were obtained from the ADNI database before October 15, 2019. According to the ADNI protocol, the criteria for MCI were: (1) subjective memory complaints; (2) objective memory loss defined as scoring below an education-adjusted cut-off score on delayed recall of the Wechsler Memory Scale (WMS-R) logical memory test; (3) a global Clinical Dementia Rating score of 0.5; (4) a Mini-mental State Examination (MMSE) score of equal to or higher than 24 out of 30; and (5) general cognitive and functional performance sufficiently preserved such that a diagnosis of dementia could not be made by the site physician at the time of screening. Meanwhile, the criteria for HC were: (1) an MMSE score of equal to or higher than 24 out of 30; (2) a Clinical Dementia Rating score of 0; and (3) has no report of any cognition complaint. Besides, no signs of depression (geriatric depression scale, GDS score < 5) or dementia were present in all subjects. All subjects with the following clinical manifestations were excluded: (1) significant medical, neurological, or psychiatric illness; (2) a history of apparent head trauma; (3) use of non-AD-related medication known to influence cerebral function; and (4) alcohol or drug abuse. After careful screening, eventually, 61 MCI with APOE ε3/ε3, 10 MCI with APOE ε2/ε3, 73 HC with APOE ε3/ε3, and 24 HC with APOE ε2/ε3 entered the subsequent analyses (Table 1 and Supplementary Figure 1).

**TABLE 1:** Demographic characteristics and cognitive scores of the study population.

	APOE ε 3/ε 3 HC	APOE ε 2/ε 3 HC	APOE ε 3/ε 3 MCI	APOE ε 2/ε 3 MCI	F/χ 2	*p*-value
						
Number	73	24	61	10		
**Demographic characteristic**						
Age, years, mean (SD)	73.06 ± 5.81	73.04 ± 5.05	70.67 ± 6.78	70.51 ± 7.43	2.07	0.11
Gender (M/F)	28/45	11/13	32/29	7/3	5.04	0.17
Education, years, mean (SD)	16.67 ± 2.39	16.50 ± 2.55	16.21 ± 2.27	15.50 ± 2.80	0.92	0.43
**Cognitive scores**						
ADNI-MEM	1.12 ± 0.56	1.12 ± 0.56	1.14 ± 0.80	0.96 ± 0.67	21.73	0.00^abcd^
ADNI-EF	1.14 ± 0.80	1.01 ± 0.68	0.45 ± 0.95	0.62 ± 0.69	8.12	0.00^abc^
ADNI-LAN	0.96 ± 0.67	0.87 ± 0.66	0.34 ± 0.76	0.57 ± 0.65	9.13	0.00^abc^
ADNI-VS	0.27 ± 0.55	0.28 ± 0.57	−0.40 ± 0.71	−0.88 ± 0.92	3.55	0.16^b^

*Data are presented as means ± standard deviations. HC, healthy control; MCI, mild cognitive impairment; ADNI-MEM, Memory; ADNI-EF, executive function; ADNI-LAN, language; ADNI-VS, visuospatial function. ^a–c^post hoc analysis further revealed the source of ANOVA difference, p < 0.05 (^a^among four groups; ^b^HC APOE ε3/ε3 vs MCI APOE ε3/ε3; ^c^HC APOE ε2/ε3 vs MCI ε3/ε3; d HC APOE ε3/ε3 vs MCI APOE ε2/ε3).*

### Neuropsychological Assessment and APOE Genotyping

All subjects underwent extensive neuropsychological batteries to assess their general mental status and other cognitive domains. We used composite scores for executive functioning (ADNI-EF), memory (ADNI-MEM), language (ADNI-LAN), and visuospatial function (ADNI-VS). All these scores have been validated in published papers (Crane et al., 2012; Gibbons et al., 2012). APOE genotyping for all participants was performed as previously described (Saykin et al., 2010). Briefly, APOE genotyping for all subjects was performed using the DNA extracted from peripheral blood cells. The cells were collected in single EDTA plastic tubes (10 ml) and were sent via overnight delivery, at room temperature, to the University of Pennsylvania AD Biofluid Bank Laboratory.

### Data Acquisition

All subjects were scanned using the 3.0-Tesla MRI scanners for specific scanner types and the number of subjects scanned by which details could be seen in the Supplementary Material (Supplementary Figure 3). Structural images were acquired using a 3D MPRAGE T1-weighted sequence with the following parameters: echo time (TE) = 2.98 ms; repetition time (TR) = 2,300 ms; 170 sagittal slices; inversion time (TI) = 900 ms; within plane FOV = 256 mm × 240 mm; flip angle = 9°; voxel size = 1.1 mm × 1.1 mm × 1.2 mm; band width = 240 Hz/pix. The rsfMRI images were acquired using an echo-planar imaging sequence with the following parameters: 140 time points; TE = 30 ms; TR = 3,000 ms; flip angle = 80°; number of slices = 48; slice thickness = 3.3 mm; matrix = 64 × 64; and spatial resolution = 3.31 mm × 3.31 mm × 3.31 mm. In accordance with the ADNI database’s human scan protocol, all participants kept their eyes open with fixation during the entire rsfMRI scan.

We downloaded the 18F FDG-PET data from the ADNI database in their most processed formats (Coreg, Avg, Standardized Image, and Voxel Size). The time interval between the scanning of PET and MRI was under 12 months. The standardized uptake value ratio (SUVR) of PET were also downloaded from the ADNI. Notably, FDG-PET data were not available for some of the subjects in the current study. Thus, FDG-PET data included in the current study consists of eight out of 10 of MCI (80.0%) with APOE ε2/ε3, 59 out of 61 MCI (96.7%) with APOE ε3/ε3, seven out of 24 HC (29.2%) with APOE ε2/ε3, and 32 out of 73 HC (43.8%) with APOE ε2/ε3.

### Imaging Pre-processing

We pre-processed the rsfMRI data using the DPABI (Yan et al., 2016) with SPM12 on the MATLAB platform (MathWorks, Natick, MA, United States). The first 10 time points of the rsfMRI data were discarded due to the instability of the initial MRI signal and the subjects’ adaptation to the scanning noise. The remaining 130 images were corrected for both timing differences between each slice and head motion (Friston 24 parameters). Subjects with more than 2.0 mm maximum displacement in any of the *x*, *y*, or *z* directions or 2.0° of any angular motion during the whole scan were discarded. We then co-registered the T1-weighted images to the mean rsfMRI image based on rigid-body transformation, spatially normalized to the Montreal Neurological Institute (MNI) space, and then resampled to 3 mm × 3 mm × 3 mm cubic voxels. Functional images were spatially smoothed with a 6 mm × 6 mm × 6 mm Gaussian kernel of full width at half maximum to decrease spatial noise. Linear trends estimation was then performed. To remove the residual effects of motion and other non-neuronal factors, Friston 24 head motion parameters, white matter signals, and cerebrospinal fluid signals were corrected as a nuisance. To remove the global signal in the pre-processing of the rsfMRI data, we omitted to regress the signal out. Finally, the fALFF was calculated as the ratio of the low-frequency power spectrum to the power spectrum of the whole frequency range. The time series was first converted to a frequency domain with a fast Fourier transformation to obtain the power spectrum to be elaborated further. The square root of the power spectrum was then computed at each frequency, and the averaged square root was obtained across 0.01–0.08 Hz at each voxel (Zou et al., 2008; Sarappa et al., 2017).

The accuracy of the PET-related analysis is limited by the partial volume effects (PVE), which affects the quantitative analysis and visual interpretation of the images. The PETPVE12 toolbox (PETPVE12: an SPM toolbox for PVE correction in brain PET, Application to amyloid imaging with FDG-PET) comes with different modules suitable for PVE-correction and quantitative analysis of PET data. To be more specific, the procedures of correction for PVE are as follows. First, the structural MRI (T1-weighted) data were segmented into Grey Matter (GM), white Matter (WM), Cerebrospinal Fluid (CSF), and skull-stripped image based on the segmentation function of the VBM8 toolbox.^2^ Second, the structural MRI (without skull-stripping) was used as “Reference” images, and FDG-PET images were used as “Source image.” Thirdly, a voxel-based method was performed using the 3-compartmental algorithm including GM, WM, and CSF, which is described as Müller-Gärtner et al. (1992) (MG) (Müller-Gärtner et al., 1992) or “modified Müller-Gärtner” – mMG (Rousset et al., 1998) to correct for the PVE of the PET images.

### Statistical Analysis

#### Demographic Analyses

Quantitative variables are expressed as the mean and standard deviation. The categorical variables are given as absolute and relative frequencies. All statistical analyses were performed using the IBM SPSS20 statistical software for Windows. Regarding the demographics, the Chi-square test was used for gender distribution difference assessment (*p* < 0.05). We then used the analysis of variance (ANOVA) to compare the education, age, and neuropsychological scales among all groups. *Post hoc* analysis of two-sample *t*-test was performed afterward (Bonferroni corrected, *p* < 0.05).

#### Imaging Analyses

The statistical analyses of imaging data were conducted using the DPABI toolbox. Specifically, we performed a 2 × 2 mixed effect analysis and explored the main effect of APOE (ε2/ε3 carriers VS. ε3 homozygotes) and cognitive status (NC vs. MCI). The potential interaction effects between APOE (ε2/ε3 carriers vs. ε3 homozygotes) and cognitive status (NC and MCI) were investigated as well. To control the effect of cortical atrophy on the functional analysis, normalized modulated (with the volumetric information encoded) GM maps were used as covariate images (*p* < 0.01, cluster level < 0.05, GRF correction), which could partly cut down the significance of group differences (Han et al., 2011). To further understand how APOE and cognitive status interacted on regional brain activities, we extracted the mean fALFF values from the significant cluster (IPL) and performed *post hoc* pair wise comparisons (*p* < 0.05, Bonferroni correction). To explore the clinical significance of imaging metrics, we then correlated the mean fALFF with neuropsychological scales.

Also, we investigated the relationships between the imaging metrics and neuropsychological measurement for the four groups, respectively. It also should be noted that the correlations were performed only within the regions exhibiting significant differences between groups (*p* < 0.005, uncorrected).

## Results

### Demographic and Clinical Characteristics

There are no statistically significant differences in gender, age, or education among the four groups (p > 0.05). Additionally, there were substantial differences among groups in some comprehensive neuropsychological scores, such as ADNI-EF, ADNI-MEM, and ADNI-LAN. However, no differences in the ADNI-VS among the four groups were observed. Detailed information can be found in Table 1.

### fALFF Analyses

**FIGURE 1:**
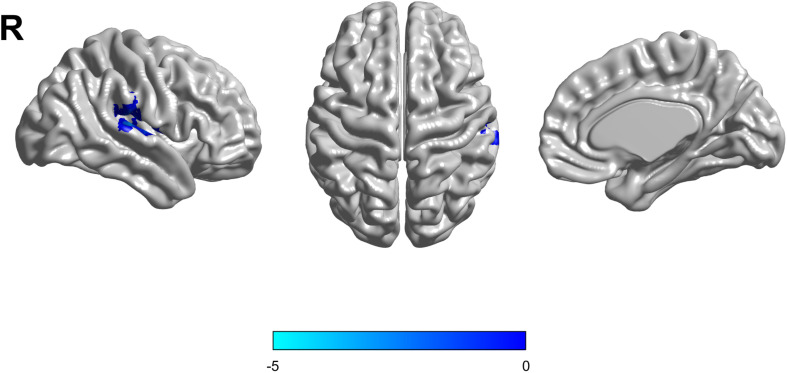
The decreased fractional amplitude low-frequency fluctuations (fALFF) was found in the transverse temporal gyrus in the APOE ε2/ε3 carriers groups compared to the APOE ε3/ε3 carriers’ groups. The statistical threshold was set at *p* < 0.01 with a cluster-level *p* < 0.05 (two-tailed, GRF corrected).

**FIGURE 2:**
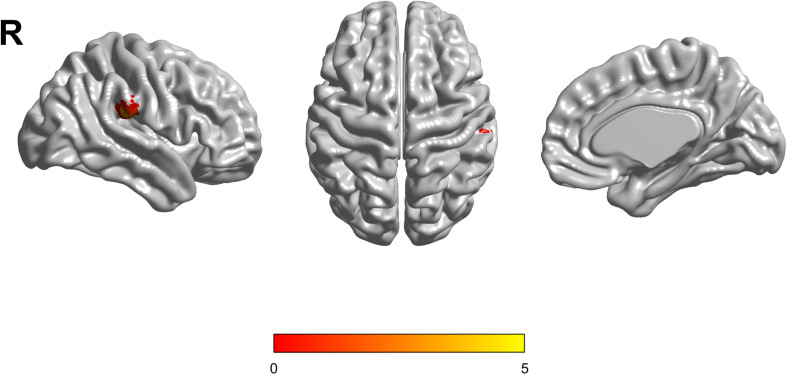
The increased fractional amplitude low-frequency fluctuations (fALFF) were found in the inferior parietal lobule (IPL) among healthy controls (HC) with APOE ε3/ε3, HC with APOE ε2/ε3, mild cognitive impairment (MCI) with APOE ε3/ε3, and MCI with APOE ε2/ε3. The results were obtained by analysis of covariance (ANCOVA) analysis adjusted with GM [*p* < 0.01, cluster level < 0.05, two-tailed, Gaussian random field (GRF) correction].

**FIGURE 3:**
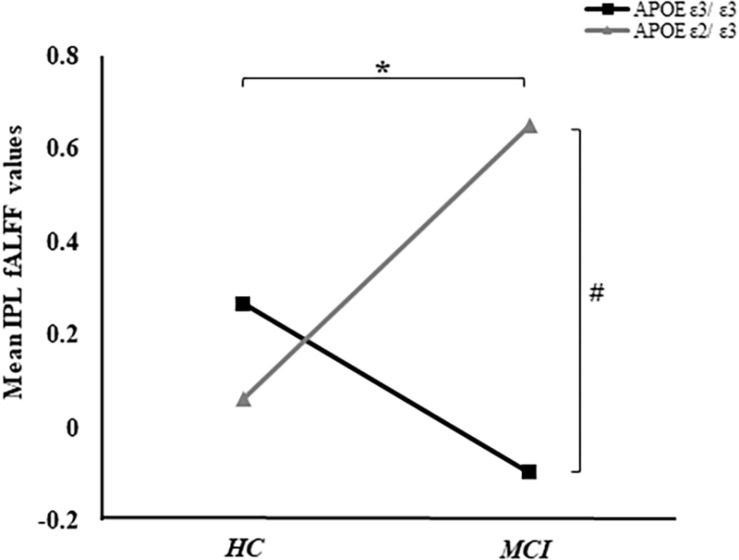
The “APOE × disease” (interaction) effects between APOE and cognitive status were shown. In addition, the average fALFF values in the IPL of groups were demonstrated. *Represents *p* < 0.05 between MCI and HC. ^#^Represents *p* < 0.05 between APOE ε3/ε3 and APOE ε2/ε3 in MCI.

Based on the mixed-effects analysis, we computed three statistical maps which generated: (1) a t-map showing the main effect of APOE, and the effect of the APOE ε2 allele found that ε2 carriers had a decreased fALFF in the transverse temporal gyrus than non-carriers in the MCI groups (*p* < 0.01, cluster level < 0.05, GRF correction) (Figure 1); (2) a t-map showing the main effect of cognitive status, and the results showed that MCI had a lower fALFF than the HC group in IPL (*p* < 0.01, cluster level < 0.05, GRF correction); and (3) an F-map showing the interaction between APOE and cognitive status. In addition, the “APOE × disease” (interaction) effects are located in the inferior parietal lobule (IPL; *p* < 0.01, cluster level < 0.05, GRF correction) (Figures 2, 3). We summarized the details of the brain regions with a difference in spontaneous activity (Table 2). Our results of the *post hoc* analyses (Bonferroni correction, *p* < 0.05) of fALFF values in IPL showed that the subjects with APOE ε2/ε3 carriers had an increased fALFF values than APOE ε3/ε3 in the MCI groups (Figure 3).

**TABLE 2:** Analysis of the covariance (ANCOVA) results across the four groups.

	Brain region	Peak MNI coordinate			Peak intensity	Number of voxels
		X	Y	Z		
Interaction Effect	IPL	51	−27	24	22.884	15
Genotype Effect	Transverse Temporal Gyrus	51	−21	15	−4.434	24
Disease Effect	Frontal Lobe	6	54	−3	−3.771	54

*IPL, inferior parietal lobe.*

### Correlations Between Neuropsychological Tests and fALFF Values

The fALFF of IPL was negatively correlated with ADNI-VS (*r* = −0.16, *p* < 0.05). Although, there were no significant differences of ADNI-VS summary score among the four groups, we found a difference between APOE ε3 carriers NC and APOE ε3 homozygous MCI (*p* = 0.029) (Table 1). Moreover, no significant relationships between IPL and ADNI-MEM, ADNI-EF, and ADNI-LAN were found. We also calculated the average fALFF values in IPL and average ADNI-VS scores of the four groups (Supplementary Table 1 and Figure 3).

### FDG-PET Analysis

**FIGURE 4:**
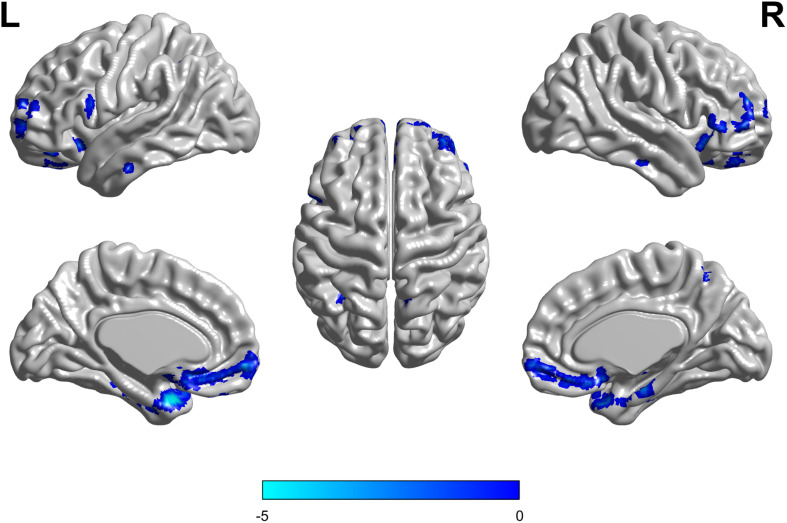
The APOE ε2/ε3 carriers groups had a slight decreased standard uptake value ratio (SUVR) of FDG-PET in the Frontal Lobe compared to the APOE ε3/ε3 homozygote groups. The statistical threshold was set at *p* < 0.01 with a cluster-level of *p* < 0.05 (two-tailed, GRF corrected).

There were no interaction effects between the APOE genotype and disease status among the four groups in the FDG PET mapping. As for the effect of the genotype, we found that APOE ε3/ε3 carriers had a higher frontal lobe SUVR than APOE ε2/ε3 carriers (GRF corrected, threshold *p* < 0.01 with cluster-level *p* < 0.05, two-tailed; Figure 4). We did not find any difference between HC and MCI as for the effect of the group.

## Discussion

We investigated the interaction effects between the APOE ε2 alleles versus disease status on intrinsic brain activity and metabolism using the fALFF and FDG-PET mapping, respectively. Our main findings include: (1) the “APOE × disease” (interaction) effects on regional spontaneous brain activity were located in the IPL; (2) APOE ε2 carriers had a lower fALFF in the transverse temporal gyrus and frontal lobe SUVR than non-carriers. Our study may provide original insights into the understanding of the impact of APOE ε2 on MCI pathologies.

### Interaction Effect of APOE ε2 Alleles and MCI on fALFF Was Located in the Inferior Parietal Lobe

We found that the interaction effects of fALFF were located in the IPL. In addition, the fALFF of IPL was negatively correlated with ADNI-VS (*r* = −0.16, *p* < 0.05). Functionally, IPL is involved in the visuospatial function, sensory-motor processing (Iacoboni, 2005; Keysers and Gazzola, 2009), executive control (Uddin et al., 2011), and salience detection (Seeley et al., 2007). Previous studies demonstrated that the atrophy of IPL was related to a worse cognitive status (Keilp et al., 1996) and AD progression (Whitwell et al., 2008). Autopsy studies reported that the IPL is susceptible to amyloid plaques and neurofibrillary tangles in MCI and AD patients (Braak and Braak, 1991; Markesbery et al., 2006; Nelson et al., 2009). Studies also showed that the combination of metabolic rates in the IPL and genetic risks (APOE ε4 carriers) could predict the cognitive decline for preclinical AD detection (Small et al., 2000). Moreover, the presence of the APOE ε4 allele is linked to a worse visuospatial working memory (Goltermann et al., 2019). In conclusion, our results suggested that the APOE ε2 allele influences the regional brain spontaneous activity patterns in MCI. Furthermore, numerous previous studies have consistently reported a protective effect of APOE ε2, manifested as promoting the clearance and degradation of Aβ (Jiang et al., 2008), regulating neuroinflammation, and fighting against gliosis (Miyata and Smith, 1996; Mahley et al., 2009) and the slowing episodic memory decline (Helkala et al., 1996). In accordance with our study, the APOE ε2 allele might decelerate the disease progression in IPL associated with visuospatial working memory in MCI.

### APOE ε2 Carriers Showed a Decreased Brain Activity and Metabolism Merely in MCI Patients

Our analyses showed that APOE ε2 carriers had an increased fALFF value than MCI non-carriers in the IPL. The possible explanation behind this might be the recessive pathological influence in healthy subjects. We suspected that the APOE ε2 gene showed the protective effect after the appearance of cognitive impairment. For group comparison analysis (effects of cognitive status), our analysis in the IPL showed that MCI had a lower fALFF than the HC groups. With the increasing disease burden, some regions are exposed to the AD-related pathology and perceive the abnormal intensity of fluctuations. The different activation of other areas may not induce a sufficient brain activity intensity (Yang et al., 2018). The changes in brain activity suggested the pathological progression of MCI. Our results are consistent with the previous studies that different brain activity patterns evaluated by fALFF alterations provide evidence for the disease’s progressive pathology (Rasero et al., 2017; Yang et al., 2018).

Regarding the effects of the APOE ε2 allele, we found that ε2 carriers had a decreased fALFF in the transverse temporal gyrus compared with the ε3 homozygote. Of note, cognitive impairment, only at an early stage, could be seen in MCI. Consequently, the brain might take countermeasures to compensate for the dysfunctions caused by the pathological injury. This revealed that ε2 carriers only need relatively lower brain activities to compensate for the AD-related pathological damage and maintain cognitive wellbeing. Notably, our assumption is also further supported by our FDG-PET analysis. We found that APOE ε2 carriers had a lower frontal lobe SUVR values than non-carriers. The FDG-PET to reflect the metabolic alternation (Hattori, 1987; Vergallo et al., 2021; Yu et al., 2021). The FDG-PET results have the similar trends with our rsfMRI results, suggesting that APOE ε2 carriers in MCI patients might need fewer brain activities to cope with AD-related pathologies. Interestingly, no interaction effects of SUVR of the APOE ε2 allele and disease status existed among the four groups, which may be partly explained by the small samples of the FDG-PET data.

Previous studies also showed that APOE ε2 was associated with an increased neuropathology and decreased risk of dementia (Berlau et al., 2009). Given the previous evidence of the protective effects of APOE ε2, our results provided evidence for understanding the mechanisms through which the APOE ε2 allele affects the pathological progression in MCI. Multiple studies show that APOE ε2 carriers had a more effective way to clear amyloid deposition (Berlau et al., 2009; Serrano-Pozo et al., 2015). Moreover, the APOE ε2 is against the formation of neuritic plaques and the spreading of neurofibrillary tangles (Serrano-Pozo et al., 2015). We thus speculate that the APOE ε2 allele might exert a protective effect in analogy with a higher cognitive reserve and increase the tolerance of AD pathology (Stern, 2012). Similarly, MCI APOE ε2 carriers tend to have a more severe pathology than HC, representing an attempt for compensatory response to MCI pathology. Furthermore, previous neuroimaging evidence demonstrated an increased functional connectivity (FC) of the entorhinal cortex (ERC) network, which suggested a compensatory effect of the APOE ε2 alleles (Das et al., 2013; Chen et al., 2016).

### A Negative Correlation Was Found Between the rsfMRI Metrics and Visual-Spatial Scores

The fALFF of the IPL was negatively correlated with the visuospatial function in all four groups. Then, we calculated the average IPL in the four groups. MCI with APOE ε2 carriers has the highest IPL value and the worst visual-spatial function than others (Supplementary Table 1 and Figure 2). Previous studies indicated that neuronal hyperexcitability at an early stage of AD has been an increasingly observed phenomenon (Palop et al., 2007; Bero et al., 2011; Busche and Konnerth, 2015; Palop and Mucke, 2016; Sosulina et al., 2021). The increased fALFF values may not stand for absolute protection but might be the outcomes of the hyperactivity of a neuron which could compensate for the dysfunction to some extent. We thought that an increased cerebral activity may play a protective role against the pathological processes (amyloid deposition and neurofibrillary tangles) but these effects were not enough for the maintenance of cognitive function. In the HC groups, APOE ε2 carriers had relatively lower average fALFF values in the IPL but a slightly higher visual-spatial score than non-carriers. Combined with the previous studies which observed that the APOE ε2 gene could promote the clearance and degradation of Aβ (Jiang et al., 2008), regulate neuroinflammation, and fight against gliosis (Miyata and Smith, 1996; Mahley et al., 2009). These mechanisms might be suggestive of the protective effect of the APOE ε2 allele in the HC population. Consistent with other studies, these results indicated a protective effect of the compensation mechanism. There were no associations between the imaging metrics and other neuropsychological test scores, which might explain the relatively small cognitive differences between MCI and HC. Thus, the effect of the APOE ε2 allele was not significant among the four groups. Considering the small sample size of MCI APOE ε2 carriers, our current results should be interpreted cautiously.

We took together the neuroimaging and neuropsychological scores results, suggesting that the APOE ε2 allele plays a protective role in a compensatory mechanism. This compensatory effect maintained the normal functioning of brain activities despite the neuropathological changes in MCI.

## Limitations

There are several limitations to our studies. First, the sample size of MCI with APOE ε2 carriers was relatively small compared to the other three groups, which makes the population unevenly distributed in each group. Epidemiologically, APOE2 homozygotes comprise <1% of the general population (Farrer et al., 1997; Coon et al., 2007). Thus, it is challenging to gather these subjects, and overestimating these subjects’ prevalence may affect our study results. Furthermore, the current results should be taken with caution due to the limited number of samples, and future replications with the separate cohort and a sufficient number of sample size are warranted. Second, not all participants underwent both the resting-state functional MRI and FDG-PET, and it resulted in less participants in the FDG-PET study, which might weaken the statistical effects to some extent. Third, the standards-compliant data which we selected from the ADNI only consist of 140-time points. A recent publication from the Gagan Wig group showed that at least 600 sample points (scanning time more than 10 min) are necessary to produce a reliable and robust signal (National Academies of Sciences, Engineering, and Medicine, 2020). We nevertheless repeated the experiment without removing the 10-time points, and the results of the interactive effect were mostly unchanged (Supplementary Figure 3). Despite all these, given that the ADNI is a relatively reliable dataset and numerous studies have been published based on the ADNI, our results are sound to some extent. Finally, our cross-sectional study failed to assess the APOE ε2 allele’s long-term effects during the progression of MCI. Thus, longitudinal studies are needed to explore the impact of genotype in brain activity patterns for the AD continuum, from healthy aging to more advanced clinical stages. In future studies, we will conduct research using neuroimaging methods to evaluate APOE ε2 during the transformation from healthy people to MCI and finally conversing with AD.

## Conclusion

Our study supports the idea that the APOE ε2 is closely linked to the AD progression’s protective role. APOE ε2 carriers might play a protective role in transforming MCI to AD through a compensatory mechanism. Also, our findings would potentially serve as functional metrics for risk stratification. As our results showed, the APOE ε2 allele indeed alters the brain activity patterns. The current study is carried out in healthy subjects and MCI, which may reveal the role of APOE ε2 in cognitive decline. Different cognitive stages could be identified in patients with a biological AD, whether the APOE ε2 allele exerts distinct pathological effects and demonstrates unique neuroimaging performance is still worth exploring.

## Data Availability Statement

The datasets generated and/or analyzed during this study are available in the ADNI study. More details are in www.adni-info.org.

## Ethics Statement

All procedures performed in studies involving human participants were in accordance with the ethical standards of the institutional and/or national research committee and with the 1964 Helsinki declaration and its later amendments or comparable ethical standards. Written informed consent was obtained from all participants and/or authorized representatives and the study partners before any protocol-specific procedures were carried out in the ADNI study.

## Author Contributions

XLi and QZ designed the study and wrote the first draft of the manuscript. XLu analyzed the MRI data and wrote the protocol. XLi and QZ collected the clinical and MRI data. KL, HH, SW, XG, JW, RZ, TZ, ZL, YF, XX, PH, and MZ assisted with the research design and interpretation of results. All authors contributed to the final manuscript and read and approved the final manuscript.

## Conflict of Interest

The authors declare that the research was conducted in the absence of any commercial or financial relationships that could be construed as a potential conflict of interest.

## Footnotes

1 http://adni.loni.usc.edu/

2 http://dbm.neuro.uni-jena.de/wordpress/vbm/
