# Supplementary material for: Effects of APOE ε2 on the Fractional Amplitude of Low-Frequency Fluctuation in Mild Cognitive Impairment: A Study Based on the Resting-State Functional MRI
Source: Front Aging Neurosci. 2021 Apr 29;13:591347. doi: 10.3389/fnagi.2021.591347 (PMC8117101; doi:10.3389/fnagi.2021.591347)

Supplementary material

Supplementary Table 1

The supplementary table 1 showed the average fALFF values in IPL and average ADNI-VS scores of four groups.

	APOE ε3/ε3 NC	APOE ε2/ε3 NC	APOE ε3/ε3 MCI	APOE ε2/ε3 MCI
IPL, mean	0.27	0.06	-0.98	0.65
ADNI-VS, mean	0.28	0.29	-0.40	-0.89

Abbreviation:

IPL: Inferior parietal lobe,

ADNI-VS: visuospatial function

Supplementary Table 2

Supplementary Table 2 shows specific scanner types, and the number of subjects scanned, which can be seen in Supplementary Material (Supplementary Table 2). Chi-square test showed no difference among groups (χ^2=10.872, P=0.092).

	Total number	GE Medical Systems	SIEMENS	Philips Medical Systems
APOE ε3/ε3 NC	74	20	27	27
APOE ε2/ε3 NC	24	3	12	9
APOE ε3/ε3 MCI	61	10	15	36
APOE ε2/ε3 MCI	10	3	3	4

Supplementary Figure 1 Schematic of the data analysis pipeline. We recruited subjects from the ADNI database before October 15, 2019. 168 subjects were included after a careful screening procedure. Furthermore, SUVR was extracted from FDG-PET among subjects who had this examination. Abbreviation: resting-state MRI, resting-state magnetic resonance imaging; HC, healthy controls; MCI, mild cognitive impairment; IPL, inferior parietal lobule; ANCOVA, analysis of covariance; SUVR, standard uptake value ratio (SUVR).

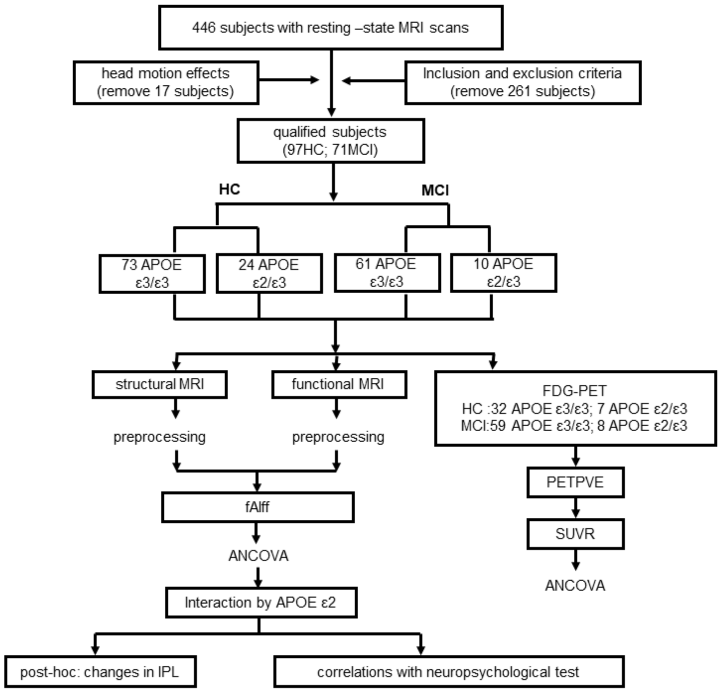

Supplementary Figure 2 illustrates the average fALFF values in IPL and average ADNI-VS scores of four groups.

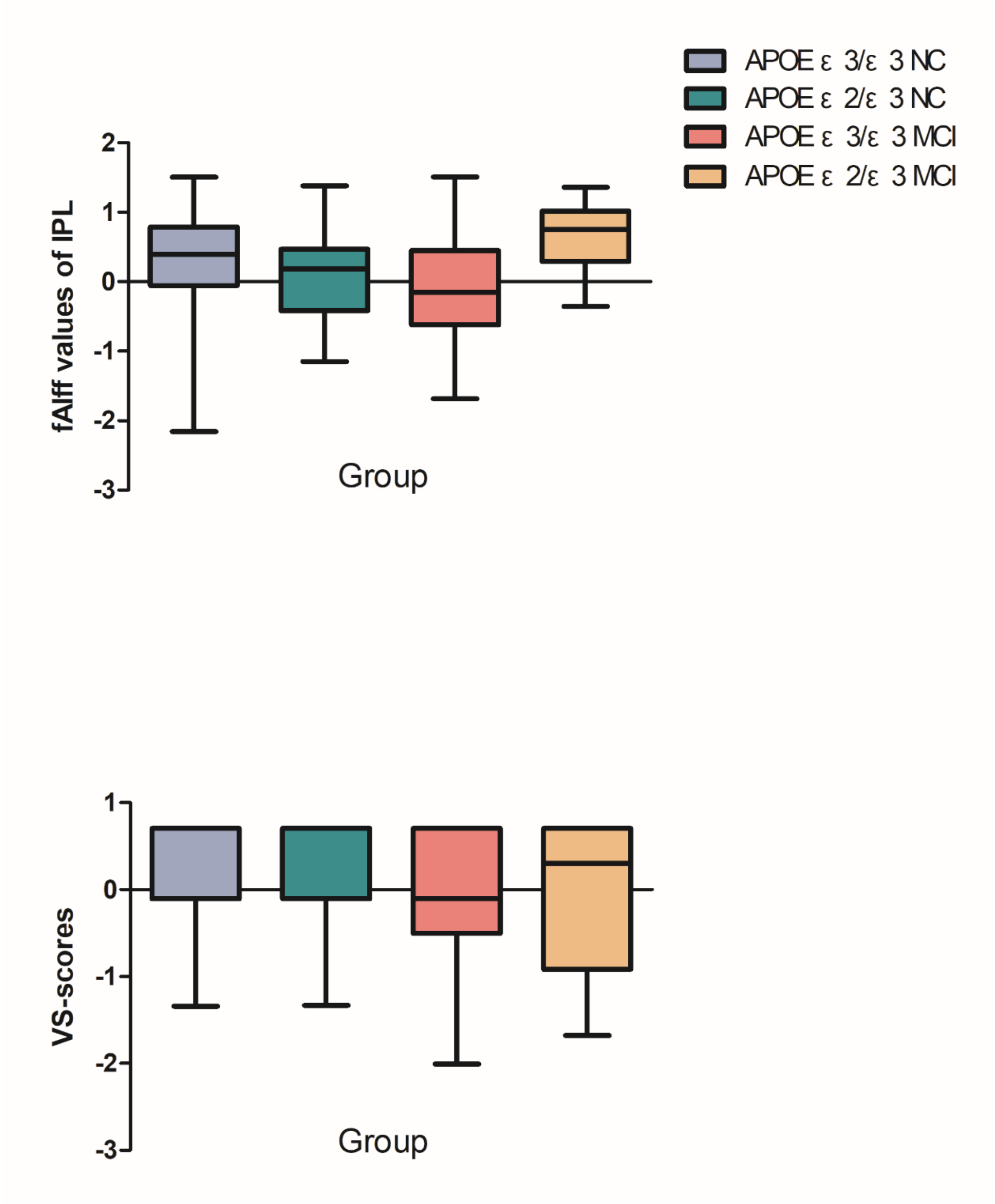

Supplementary Figure 3 Figure A showed interactive effect results without removing the first 10 time points, which are located in Precuneus. Figure B showed the result of the interactive effect with removing the initial 10-time points, which are located in the inferior parietal lobule (IPL). (GRF corrected, threshold $p < 0.01$ with cluster-level $p < 0.05$, two-tailed).

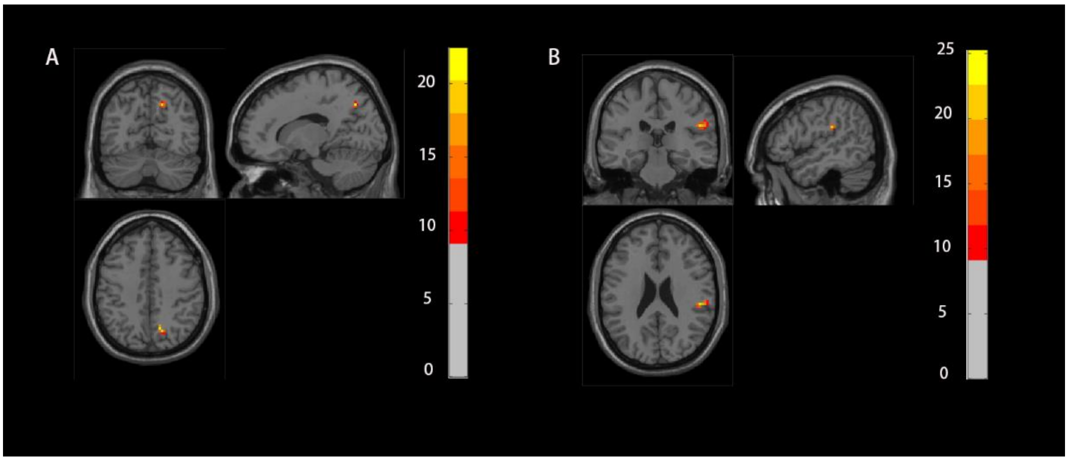

Supplement: Supplementary file 1 [file Data_Sheet_1.PDF]
